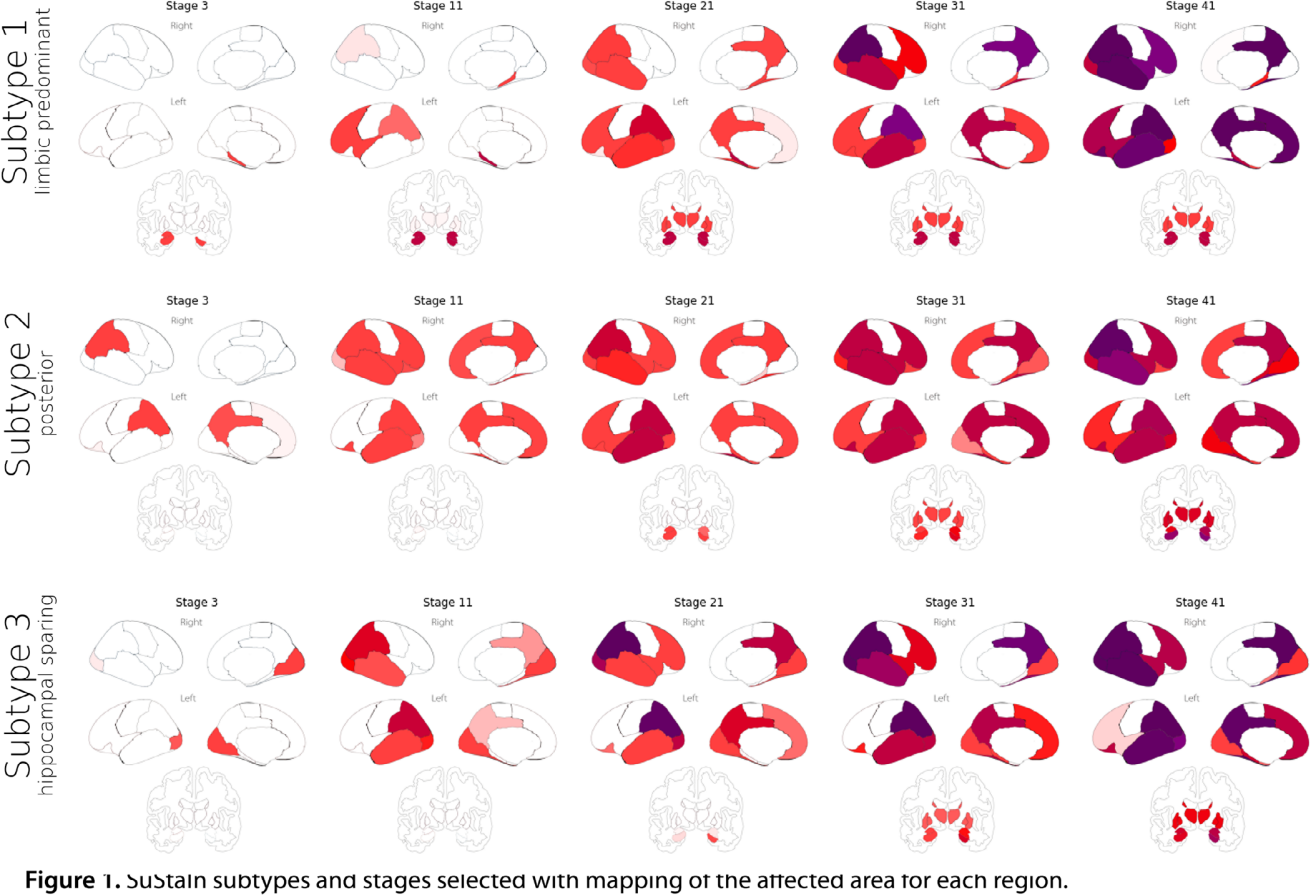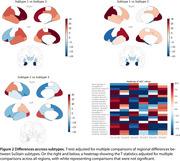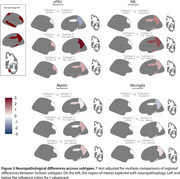# Characterization of MRI‐derived atrophy subtypes using post‐mortem MRI and neuropathological markers in Alzheimer's disease

**DOI:** 10.1002/alz70856_106454

**Published:** 2026-01-11

**Authors:** Ismael Luis Calandri, Laura E. Jonkman, Sophie E. Mastenbroek, Alex J. Wesseling, Colin Groot, Frederik Barkhof, Yolande A.L. Pijnenburg, Rik Ossenkoppele

**Affiliations:** ^1^ Alzheimer center, VUMC, Amsterdam, Netherlands; ^2^ Fleni, Buenos Aires, Buenos Aires, Argentina; ^3^ Department of Anatomy and Neurosciences, Amsterdam UMC, Vrije Universiteit Amsterdam, Amsterdam Neuroscience, Amsterdam, Netherlands; ^4^ Clinical Memory Research Unit, Department of Clinical Sciences Malmö, Faculty of Medicine, Lund University, Lund, Sweden; ^5^ Amsterdam UMC, location VUmc, Amsterdam, Netherlands; ^6^ Alzheimer Center Amsterdam, Neurology, Vrije Universiteit Amsterdam, Amsterdam UMC location VUmc, Amsterdam, Netherlands; ^7^ Amsterdam UMC, location VUmc, Amsterdam, Noord‐Holland, Netherlands; ^8^ Alzheimer Center Amsterdam, Department of Neurology, Amsterdam UMC, location VUmc, Amsterdam, Netherlands; ^9^ VU University Medical Center, Amsterdam UMC, Amsterdam, Netherlands; ^10^ Clinical Memory Research Unit, Department of Clinical Sciences, Lund University, Lund, Sweden

## Abstract

**Background:**

Alzheimer's disease (AD) exhibits diverse neurodegeneration patterns, highlighting the need for detailed atrophy mapping to improve early diagnosis and monitoring. Although MRI scans are commonly used for *in vivo* brain assessment, the interpretation of atrophy patterns in the context of neuropathological findings has been limited. This study aims to address this gap by combining structural MRI with postmortem pathological data to assess howdata‐driven atrophy subtypes correlate with underlying AD pathology, axonal degeneration and myelin integrity.

**Method:**

We analyzed 2,029 MRI scans from cognitively impaired individuals with positive AD biomarkers. Cortical volumes and thicknesses were computed using FreeSurfer and harmonized across MRI scanners with NeuroCombat. Measurements were normalized using data from 620 cognitively unimpaired individuals with negative AD biomarkers. A SuStaIn (Subtype and staging inference) model was trained with detection thresholds of 0.5, 1, and 2 SDs. To validate, the trained model was used to classify and stage a subset with available postmortem MRI and neuropathological data (*n* = 34 AD, *n* = 16 controls). We quantified amyloid (4G8), pTau (AT8), NfL, myelin (PLP), and microglia (IBA1) in the fusiform gyrus, superior parietal lobule, precuneus, middle temporal gyrus, middle frontal gyrus, posterior cingulate gyrus, parahippocampal gyrus, occipital cortex and right hippocampus. A linear mixed‐effects model tested regional neuropathological differences across subtypes, adjusting for stage.

**Result:**

We found three subtypes: Subtype‐1,(limbic) initially hippocampal involvement, accounting for 55.8% of cases; subtype‐2 (posterior), initial involvement of the parietal lobes, (29.4%); and subtype‐3 (hippocampal sparing), relative preserved hippocampi until advanced stages (14.7%, Figures 1 and 2). Subtypes 1 and 2 showed significantly greater pTau deposition in middle frontal gyrus and temporal (fusiform gyrus, medial and parahippocampal) regions compared to subtype 3 (*p* <0.01, Figure 3). Subtype‐2 exhibited higher Tau burden than subtype‐1 in parietal regions (*p* <0.01). Additionally, subtype 1 had higher NfL levels in the superior parietal lobule (*p* = 0.02) than subtype‐2. No significant differences were found in amyloid and microglia distribution.

**Conclusion:**

We identified three subtypes of atrophy progression that resemble clinical observations. The distribution of post‐mortem markers generally mirrored the atrophy pattern observed across subtypes, supporting the biological plausibility of the identified subtypes.